# Prevalence of tobacco related chronic diseases and its role in smoking cessation among smokers in a rural area of Shanghai, China: a cross sectional study

**DOI:** 10.1186/s12889-019-7110-9

**Published:** 2019-06-13

**Authors:** Ruiping Wang, Yonggen Jiang, Chunxia Yao, Meiying Zhu, Qi Zhao, Limei Huang, Guimin Wang, Ying Guan, Engelgau Michael, Genming Zhao

**Affiliations:** 1 YueYang Hospital, Shanghai University of Traditional Chinese Medicine, 110 Ganhe Road, Hongkou District, Shanghai, 200437 China; 2 Songjiang Center for Disease Control and Prevention, Shanghai, China; 3 School of Public Health, Fudan University, Shanghai, China; 4 Center for Disease Control and Prevention, Atlanta, GA USA

**Keywords:** Tobacco related chronic diseases, Prevalence, Smoking cessation, Current smoker, Ex-smoker

## Abstract

**Background:**

Tobacco smoking is a recognized risk factor for many chronic diseases and previous study evidences have indicated that smokers receive smoking cessation service after the diagnosis of chronic diseases increases successful rate in quitting. But the prevalence of tobacco related chronic diseases (TCD) among smokers, as well as the role of TCD diagnosis in smoking cessation is still unclear in China.

**Methods:**

From June 2016 to December 2017, we sampled 36, 698 residents aged over 18 years by a three stage sampling in Songjiang district, Shanghai. We conducted a cross-sectional study to understand the prevalence of TCD among smokers, and the role of TCD diagnosis in smoking cessation among ex-smokers as well as the smoking cessation attempt among current smokers.

**Results:**

Over all, the prevalence of current smoking is 19.78% (48.36% for male and 0.22% for female). 15.93% of smokers have stopped smoking successfully (1, 376/8, 636). The prevalence of ten selected TCDs among smokers range from 0.63% (Chronic Obstructive Pulmonary Disease, COPD) to 36.31% (hypertension). All of 1, 376 ex-smokers had at least one kind of TCD, and 52.33% of them stop smoking after the diagnosis of TCD, the time interval between TCD diagnosis and smoking cessation ranges from 0 to 65 years, with a median of 9 years. Smokers with TCD had higher prevalence of quit smoking, and current smokers with TCD had higher smoking cessation attempt proportion.

**Conclusions:**

The prevalence of current smoking is still very high among male residents in rural area of Shanghai, and the occurrence of TCD even non-lethal one could provide an opportunity for doctors to assist the smoking cessation among smokers.

## Background

Smoking is a practice in which substance like tobacco is burned and the resulting smoke inhaled in to be tasted and absorbed into the bloodstream [1]. Generally, smoking has negative health effect and is the single largest preventable cause of morbidity and mortality all over the world [2]. World Health Organization (WHO) estimates that over 1 billion people addict to tobacco smoking, 5 million people die from tobacco-related diseases each year, and the toll will rise to over 8 million by 2030 if the current trends continue [3, 4]. Tobacco smoking is a recognized risk factor for many chronic diseases such as chronic obstructive pulmonary disease (COPD), hypertension, cardiovascular disease, atherosclerosis, diabetes, cancer and microbial infections (respiratory tract infections, bacterial meningitis), etc. [5–7], which leads to heavy burden involving health care and economic as well as social costs in all countries [8].

China is the largest producer and biggest consumer of tobacco in the world, which produce a heavy burden of tobacco related chronic diseases (TCD). The Global Adult Survey conducted in 2010 indicates that 300 million adults in China are current smokers, with 72.40% of non-current smokers are exposed to second hand smoke (SHS), and 1 million deaths are attributed to tobacco consumption each year [7–11]. Due to the heavy disease burden of smoking, it is crucial to understand the prevalence of TCDs among smokers [12]. But currently, information of TCD prevalence among smokers in China is still unclear. Previous studies demonstrating the role of hospital smoking cessation service in promoting smoking cessation among smokers, and evidence has indicated that smokers receive smoking cessation service increases successful rate in quitting [13, 14]. Similarly, the physical health status such as pregnancy as well as diagnosis of chronic diseases (CD) also promote some smokers to stop smoking [15, 16]. But the role of TCD diagnosis in promoting smoking cessation among smokers is still unclear in China.

In this paper, we conducted a cross sectional study among residents in a rural area of Shanghai China. We aim to understand the prevalence of smoking and current smoking, the prevalence of TCD among smokers, and the role of TCD diagnosis in smoking cessation among ex-smokers as well as the smoking cessation attempt among current smokers.

## Methods

### Study population

**Fig. 1:**
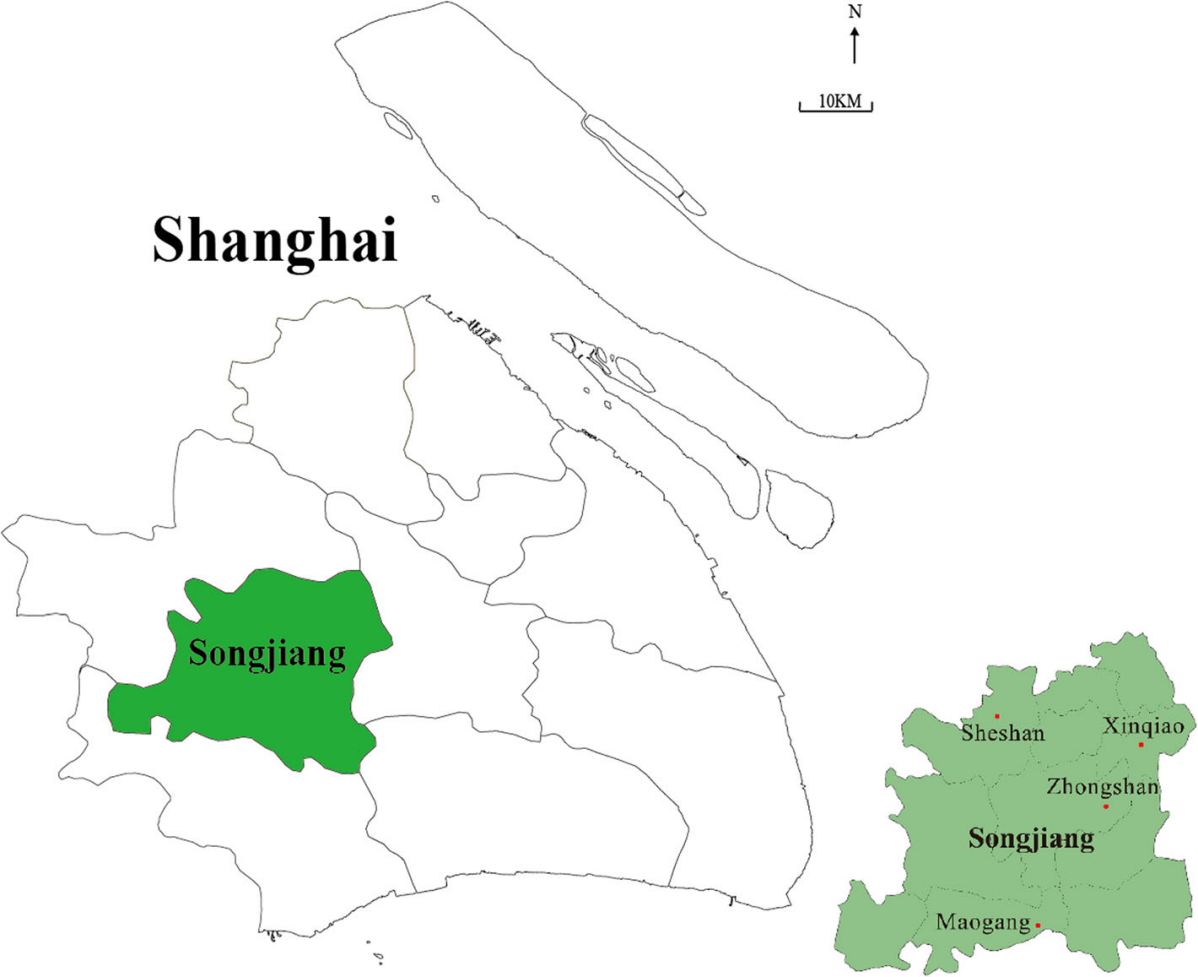
Four study sites in Songjiang district, Shanghai, China

This cross-sectional study was conducted in Songjiang District, a rural area of Shanghai from June 2016 to December 2017. Songjiang District is located in the southwest of Shanghai, with a population size of 1.76 million in the year of 2016 (Fig. 1). We employed a multistage sampling method to recruit the study population among the 15 sub-districts of Songjiang. In stage one, we randomly selected 4 out of the 15 sub-districts in Songjiang District, including Zhongshan, Xinqiao, Sheshan and Maogang (Fig. 1). In stage two, nine, eighteen, four and sixteen neighborhood committees were randomly selected from Xinqiao, Zhongshan, Sheshan and Maogang sub-district, respectively (nearly 60% of all neighborhood committees in each sub-districts). In stage 3, we recruited all individuals aged over 18 years and lived in Songjiang district for 5 years or longer from each of the 47 selected neighborhood committees. Over all, a total number of 37,543 residents were sampled and invited to participate in this study. The ethics approval was exempted by Fudan University Institution Review Board (No. IRB#6-04-0586), and an informed consent paper was signed by each participant before the questionnaire interview, finally 36, 698 residents (a response rate was 97.75%) completed the interview and were included in the final analysis.

### Data collection

Data were collected by a questionnaire designed by School of Public Health, Fudan University. The questionnaire includes four parts. Part A covered 8 demographic questions including age, gender, ethnicity, education level, monthly income, working status, marital status and occupation. Part B covered 10 tobacco related chronic diseases (TCD) information including hypertension, coronary disease, cerebral apoplexy, diabetes, chronic bronchitis, asthma, COPD, chronic gastritis, chronic hepatitis and cancer. We designed 3 questions to collect information for each TCD (e.g., *Q1 ‘Do you have hypertension*? *’, Q2 ‘What is your age when you was diagnosed as hypertension for the first time*? *’, Q3 ‘Does your family member have hypertension*? *’*). The TCD for each patients were then verified by the diseases information extracted from the local HIS (Health Information System). Part C covered 36 health-related behavior information (tobacco use, alcohol consumption, tea drink and sleep habit), we designed 16 questions to collect tobacco use information (e.g., *‘Have you ever smoked at least one cigarette every day for over six months*? *’, ‘what is your age when you smoke the first cigarette?’, ‘how many cigarettes do you usually smoke each day?’, ‘do you still smoke now?’, ‘what is your age when you quit smoking?’, ‘have you ever tried to quit smoking for over 24 hours in the recent year?’, ‘how many time do you have tried to quit smoking in the recent years?’*, etc). Part D covered personal contact information both for the investigator and the participant.

### Definition and index calculation

In this study, we define a smoker as a person who smoked at least one cigarette every day for over six months in his/her whole life-time, a current smoker is defined as a smoker who still smoke at the time of investigation, and an ex-smoker is defined as a smoker who quit smoking at the time of investigation. The prevalence of smoking is calculated as the number of smokers divided by the total number of participants, similarly, the prevalence of current smoking is calculated as the number of current smokers divided by the total number of participants. The prevalence of quit smoking is calculated as the number of ex-smokers divided by the number of smokers. The proportion of quit smoking attempt is calculated as the number of current smokers who has attempt to quit smoking in a recent year divided by the total number of current smokers. The prevalence of TCD is calculated as the number of participants who have a specific TCD divided by the total number of participants. If the age of quit smoking for an ex-smoker is smaller than the age of TCD first diagnosis, then the ex-smoker is classified into a group named ‘TCD diagnosed after quit smoking’; otherwise, the ex-smoker is classified into a group named ‘TCD diagnosed before quit smoking’. Outcome in this study is defined as ‘prevalence of quit smoking’ among smokers, and ‘proportion of smoking cessation attempt’ among current smokers. Age of participants is classified in to six age groups (‘18–29’, ‘30–39’, ‘40–49’, ‘50–59’, ‘60–69’ and ‘70–92’). Education is recorded as completed years of schooling and categorized to 5 categories of 0 year (illiterate), 1–6 years (primary school), 7–9 years (junior high school), 10–12 years (senior high school), and > 12 years (college and above). Monthly income is collected in Chinese Yuan (RMB) and classified into four groups (< 1500 RMB, 1500–2466 RMB, 2500–4999 RMB, and ≥ 5000 RMB).

### Data analysis

Data analysis was performed by SAS software (version 9.2). We described the data by using frequency counts and proportions (rate) for qualitative variables and means as well as standard deviations (SD) for quantitative variables. We applied Chi-squire test to examine the difference of smoking prevalence, current smoking prevalence and quit smoking prevalence among participants with different demographic characteristics, and the differences of TCD prevalence between non-smokers and smokers, as well as between current smokers and ex-smokers. We also applied Chi-squire test to compare the difference of quit smoking prevalence among smokers with TCD and without TCD, as well as the difference of smoking cessation attempt proportion among current smokers with TCD and without TCD. Weighted logistic regression was applied to calculated the odds ratios (OR) and 95% confidence interval (95% CI) of quit smoking prevalence among smokers with TCD compared with smokers without TCD, as well as the smoking cessation attempt proportion among current smokers with TCD compared with current smokers without TCD. Co-variables selected as potential confounders by Directed Acyclic Graph (DAG) were adjusted in the weighted logistic regression, including gender, age (age groups was changed into dummy variable and set ‘18–29’ as the reference), education (education was changed into dummy variable and set ‘illiterate’ as the reference), working status, and alcohol consumption. A box plot was produced by calculating the years for TCD diagnosed before quit smoking among ex-smokers. A *p*-value of less than 0.05 (two-tailed) was considered as statistically significant.

## Results

### Characteristics of participants

In this study, we investigated 36, 698 residents in Songjiang district, including 14, 912 males (40.63, 95% CI: (40.13–41.14%)) and 21, 786 females (59.37, 95% CI: (58.86–59.87%)). The age ranged from 18 to 92 with an average age of (56.37 ± 11.29) years old. The majority of residents were married (92.83, 95% CI: (92.57–93.10%)), and over 80% of residents had an education under junior high school (less than 12 years), more than 50% of residents’ monthly income were over 2500 RMB and 58.58% (95% CI: 58.13–58.84%) of residents were retired at the investigation time, see Table 1.

**Table 1:** The prevalence of smoking, current smoking and quit smoking among residents with different demographic characters in a rural area of Shanghai, China

Variables	Residents (36698)		Smoker (8636)		Current Smoker (7260)		Ex-smoker (1376)		Difference in smoking prevalence^★^		Difference in current smoking prevalence^★^		Difference in quit smoking prevalence^★^	
	n	(%)	n	(%)	n	(%)	n	(%)	OR	OR 95% CI	OR	OR 95% CI	OR	OR 95% CI
Gender ^€^														
Male	14,912	40.63	8571	57.48	7211	48.36	1360	15.87	1.00		1.00		1.00	
Female	21,786	59.37	65	0.30	49	0.22	16	24.62	0.002	0.002–0.003	0.002	0.002–0.003	2.45	1.35–4.44
Age (years)^€ ¥^														
18–29	1027	2.80	118	11.49	109	10.61	9	7.63	1.00		1.00		1.00	
30–39	2576	7.02	427	16.58	406	15.76	21	4.92	1.34	1.02–1.75	1.40	1.06–1.85	0.63	0.27–1.46
40–49	4600	12.53	1005	21.85	932	20.26	73	7.26	1.85	1.41–2.44	1.79	1.35–2.37	1.04	0.47–2.26
50–59	12,015	32.74	2777	23.11	2474	20.59	303	10.91	2.14	1.64–2.80	1.86	1.41–2.44	1.64	0.77–3.49
60–69	12,714	34.64	3418	26.88	2702	21.25	716	20.95	1.87	1.40–2.51	1.33	0.99–1.80	3.31	1.51–7.26
70–92	3766	10.26	891	23.66	637	16.91	254	28.51	1.31	0.97–1.77	0.85	0.63–1.16	5.02	2.27–11.12
Education^€ ¥^														
Illiterate	5392	14.69	546	10.13	455	8.44	91	16.67	1.00		1.00		1.00	
Primary	11,781	32.10	2951	25.05	2325	19.74	626	21.21	2.13	2.01–2.29	2.04	1.91–2.19	1.69	1.54–1.88
Junior High	12,973	35.35	3611	27.83	3130	24.13	481	13.32	2.04	1.91–2.21	2.03	1.90–2.19	0.84	0.65–1.08
Senior High	4298	11.71	1213	28.22	1067	24.83	146	12.04	2.34	1.79–3.10	2.16	2.01–2.37	0.77	0.57–1.03
College and above	2254	6.14	315	13.98	283	12.56	32	10.16	1.13	0.82–1.65	1.08	0.67–1.60	0.60	0.37–0.98
Marriage^€^														
Married	34,068	92.83	8233	24.17	6915	20.30	1318	16.01	1.00		1.00		1.00	
Widow/widower	1652	4.50	202	12.23	166	10.05	36	17.82	0.41	0.28–0.59	0.47	0.34–0.66	1.02	0.78–1.25
Unmarried	505	1.38	83	16.44	75	14.85	8	9.64	0.53	0.31–0.68	0.89	0.72–1.10	0.76	0.52–1.10
Divorced	473	1.29	118	24.95	104	21.99	14	11.86	0.98	0.79–1.21	1.03	0.89–1.22	0.83	0.59–1.17
Monthly income (RMB)^¥^														
< 1500	2732	7.44	615	22.51	549	20.10	66	10.73	1.00		1.00		1.00	
1500–2499	15,525	42.30	3554	22.89	3008	19.38	546	15.36	1.03	0.97–1.08	0.98	0.90–1.12	1.33	1.07–1.66
2500–4999	12,627	34.41	3076	24.36	2609	20.66	467	15.18	1.11	0.99–1.23	1.03	0.98–1.09	1.31	1.05–1.63
≥ 5000	5814	15.84	1391	23.93	1094	18.82	297	21.35	1.04	0.96–1.09	0.87	0.72–1.03	2.03	1.54–2.48
Working status^€ ¥^														
Retired	21,496	58.58	4417	20.55	3463	16.11	954	21.60	1.00		1..00		1.00	
Not retired	15,202	41.42	4219	27.75	3797	24.98	422	10.00	1.13	1.06–1.24	1.10	1.02–1.31	0.95	0.76–1.20

€: the differences between group on smoking /current smoking prevalence was statistically significant by chi-square test (*P* < 0.01)

¥: the differences between group on quit smoking prevalence was statistically significant by chi-square test (*P* < 0.01)

★: the differences between group on smoking/current smoking prevalence/quit smoking prevalence after the adjustment of covariates

### Prevalence of smoking, current smoking and quit smoking

Among 36, 698 investigated residents, 8, 636 were smokers, the prevalence of smoking was 23.53% (95% CI: 23.10–23.97%). The prevalence of smoking was significantly higher in males (57.48%) than in females (0.30%). Residents aged 18 to 29 years had the lowest prevalence of smoking, whereas residents aged 60 to 69 years had the highest prevalence of smoking, and the prevalence of smoking in other 4 age groups ranged from 16.58 to 23.66%, the prevalence of smoking was statistically significant in different age groups. Residents with education of illiterate and over college had lower prevalence of smoking than residents with education of primary and high school (junior and senior), and the difference was statistically significant. Married as well as divorced residents had higher prevalence of smoking than unmarried and widow/widower, and the differences was statistically significant. Residents with monthly income over 2500 RMB had higher smoking prevalence, but the difference was not statistically significant. Retired residents had lower smoking prevalence than non-retired residents, and the difference was statistically significant. The different prevalence of smoking in six age groups was still statistically significant, after the adjustment of gender, marriage, working status and education. And the difference in smoking prevalence among residents with different education was still statistically significant even with the adjustment of gender, working status and marriage. See Table 1.

For current smoking, the prevalence was 19.78% (95% CI: 19.38–20.19%) in 36, 698 residents. The prevalence of current smoking in male (48.36%) was significantly higher than in female (0.22%); and the prevalence of current smoking increased with the increase of age among residents. For education, marriage and working status, retired residents and residents with illiterate and above college had lower prevalence of current smoking, and married as well as divorced residents had higher prevalence of current smoking. See Table 1.

In this study, 1, 376 out of 8, 636 smokers had stopped smoking, the prevalence of quit smoking was 15.93% (95% CI: 15.16–16.71%). The prevalence of quit smoking was higher in older residents than younger residents, and retired residents had higher quit smoking prevalence than non-retired residents, the differences were all statistically significant; residents with lower education level and higher monthly income were prone to quit smoking. See Table 1.

### Prevalence of TCD

In this study, the prevalence of TCDs among 36, 698 residents ranged from 0.53% for COPD to 33.05% for hypertension. Table 2 indicated that the prevalence of TCDs ranged from 0.50 to 32.05% among non-smokers, and ranged from 0.63 to 36.31% among smokers. Chi-square test showed that the prevalence of hypertension (36.31% vs32.05%, *p* < 0.01) and chronic bronchitis (7.62% vs 7.12%, p < 0.01) among smokers were higher than non-smokers, but smokers had a lower prevalence of coronary diseases than non-smokers (3.36% vs 4.13%, *p* < 0.01). See Table 2.

**Table 2:** The prevalence of tobacco related chronic diseases among residents, and the difference between non-smokers and smokers, as well as difference between current smoker and ex-smoker, in a rural area of Shanghai, China

Tobacco related chronic diseases	No-smokes		Smoker		*P*	*P* ^*§*^	Current Smoker		Ex-smoker		*P*	*P* ^*€*^
	n	Rate (%)	n	Rate (%)			n	Rate (%)	n	Rate (%)		
Hypertension	8994	32.05	3136	36.31	< 0.01	< 0.01	2501	34.45	635	46.15	< 0.01	< 0.01
Chronic gastritis	2971	10.59	808	9.36	< 0.01	0.85	648	8.93	160	11.63	< 0.01	< 0.01
Diabetes	2225	7.93	768	8.89	< 0.01	0.82	610	8.40	158	11.48	< 0.01	< 0.01
Chronic bronchitis	1999	7.12	658	7.62	0.12	< 0.01	463	6.38	195	14.17	< 0.01	< 0.01
Coronary diseases	1159	4.13	290	3.36	< 0.01	< 0.01	218	3.00	72	5.23	< 0.01	< 0.05
Cerebral apoplexy	685	2.44	237	2.74	0.12	0.32	145	2.00	92	6.69	< 0.01	< 0.01
Chronic hepatitis	663	2.36	316	3.66	< 0.01	0.59	257	3.54	59	4.29	0.18	0.16
Asthma	582	2.07	170	1.97	0.55	0.43	120	1.65	50	3.63	< 0.01	< 0.01
Cancer	424	1.51	92	1.07	< 0.01	< 0.05	27	0.37	65	4.72	< 0.01	< 0.01
COPD	140	0.50	54	0.63	0.16	0.93	35	0.48	19	1.38	< 0.01	< 0.01

§: *P* value after the adjustment of age, gender, marriage status, working status and education

€: *P* value after the adjustment of age, working status and education

The prevalence of the ten selected TCDs ranged from 0.48 to 34.45% among current smokers, and ranged from 1.38 to 46.15% among ex-smokers. For each TCD, the prevalence among ex-smokers was higher than current smokers, and the differences of TCD prevalence between current smokers and ex-smokers were all statistically significant except for chronic hepatitis. See Table 2.

### Relationship of TCD diagnosis and smoking cessation

In this study, smokers with TCD had higher prevalence of quit smoking than smokers without TCD. Tables 3 indicated that the Odds Ratio ranged from 1.22 (95% CI: 0.91–1.63) for chronic hepatitis to 13.28 (95% CI: 8.45–20.88) for cancer. Weighted logistic regression analysis showed that smokers with TCD had higher prevalence of quit smoking after the adjustment of age, education, working status and alcohol consumption, the OR value ranged from 1.25 to 10.51. See Table 3.

**Table 3:** The prelavence of quit smoking among smokers with TCD and without TCD in a rural area of Shanghai, China

Smokers with or without TCD		Smokers who quit smoking		OR	95% CI	OR^§^	95% CI^§^
		n	Proportion (%)				
hypertension	Yes (3136)	635	20.25	1.63	1.45–1.83	1.29	1.15–1.46
	No (5500)	741	13.47	1.00		1.00	
Coronary diseases	Yes (290)	72	24.83	1.78	1.36–2.34	1.35	1.03–1.79
	No (8346)	1305	15.62	1.00		1.00	
Cerebral apoplexy	Yes (237)	92	38.82	3.52	2.69–4.60	2.47	1.88–3.26
	No (8399)	1284	15.29	1.00		1.00	
Diabetes	Yes (768)	158	20.57	1.41	1.18–1.70	1.33	1.10–1.61
	No (7868)	1218	15.48	1.00		1.00	
Chronic bronchitis	Yes (658)	195	29.64	2.42	2.02–2.89	2.06	1.72–2.48
	No (7978)	1181	14.80	1.00		1.00	
Asthma	Yes (170)	50	29.41	2.24	1.61–3.14	2.02	1.43–2.85
	No (8466)	1326	15.66	1.00		1.00	
COPD	Yes (54)	19	35.19	2.89	1.65–5.07	2.34	1.31–4.20
	No (8582)	1357	15.81	1.00		1.00	
Chronic gastritis	Yes (808)	160	19.80	1.34	1.12–1.61	1.31	1.09–1.59
	No (7828)	1216	15.53	1.00		1.00	
Chronic hepatitis	Yes (316)	59	18.67	1.22	0.91–1.63	1.25	0.93–1.68
	No (8320)	1317	15.83	1.00		1.00	
Cancer	Yes (92)	65	70.65	13.28	8.45–20.88	10.51	6.46–17.10
	No (8544)	1311	15.34	1.00		1.00	

§: OR and 95% CI after the adjustment of age, education, working status and alcohol consumption (covariates adjusted during the weighted logistic regression were selected by Directed Acyclic Graph (DAG) method)

In this study, all of 1, 376 ex-smokers had at least one kind of TCD. We divided ex-smokers into two groups according to chronological order of TCD diagnosis time and smoking cessation time. In total, 720 ex-smokers stopped smoking after the diagnosis of TCD, accounting for 52.33%. Table 4 showed that 89.83% of ex-smokers quit smoking after the diagnosis of chronic hepatitis, over 50% of ex-smokers quit smoking after the diagnosis of asthma, chronic gastritis and chronic bronchitis, and the proportion of ex-smokers who quit smoking after the diagnosis of hypertension, COPD, diabetes, coronary diseases and cerebral apoplexy ranged from 18.48 to 46.46%. See Table 4.

**Table 4:** The proportion of tobacco related chronic diseases (TCD) that diagnosed before and after smoking cessation among ex-smokers in a rural area of Shanghai, China

Tobacco related chronic diseases (TCD)	TCD diagnosed before smoking cessation		TCD diagnosed after smoking cessation		*N = 1376*
	n	Proportion (%)	n	Proportion (%)	
Chronic hepatitis	53	89.83	6	10.17	59
Asthma	35	70.00	15	30.00	50
Chronic gastritis	105	65.63	55	34.38	160
Chronic bronchitis	120	61.54	75	38.46	195
Hypertension	295	46.46	340	53.54	635
COPD	7	36.84	12	63.16	19
Diabetes	58	36.71	100	63.29	158
Coronary diseases	26	36.11	46	63.89	72
Cerebral apoplexy	17	18.48	75	81.52	92
Cancer	4	6.15	61	93.85	65

**Fig. 2:**
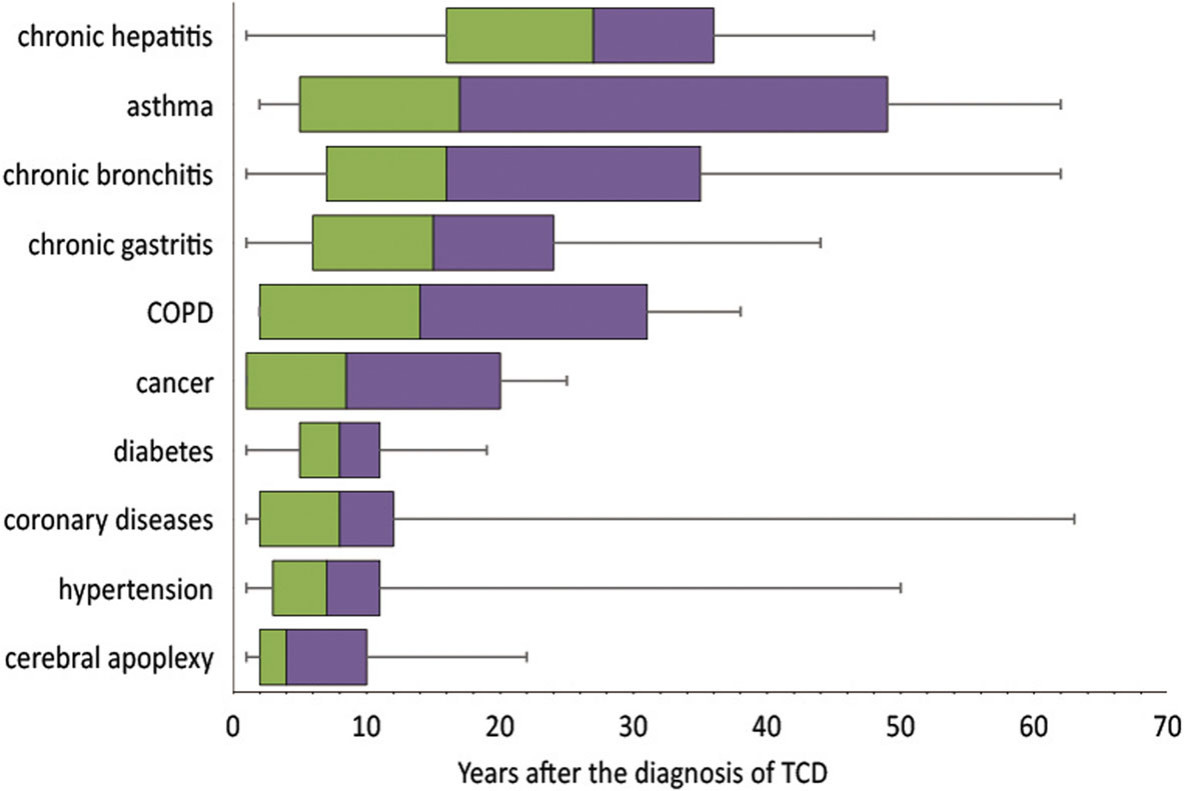
The time interval (P0, P25, P50, P75, P100) between TCD diagnosis and smoking cessation among ex-smoker in a rural area of Shanghai, China

Figure 2 indicated that 50% ex-smokers quit smoking in 4 years after the diagnosis of cerebral apoplexy, over 50% ex-smokers stopped smoking in 8 years after the diagnosis of diabetes, hypertension, cancer and coronary diseases, and 50% ex-smokers quit smoking in 16 years after the diagnosis of COPD, chronic gastritis, chronic bronchitis, asthma and chronic hepatitis. But for ex-smokers with chronic hepatitis, 50% quit smoking in 30 years after the diagnosis. See Fig. 2.

### Smoking cessation attempt among current smokers

In this study, current smokers with hypertension (14.83% vs 15.68%) as well as cancer (14.81% vs 15.39%) had slightly lower smoking cessation attempt proportion than current smokers without the TCD, but was not statistically significant. For coronary diseases, cerebral apoplexy, diabetes, chronic bronchitis, asthma, COPD, chronic gastritis and chronic hepatitis, current smokers with these TCDs had statistically significant higher smoking cessation attempt proportion than current smokers without these TCD after the adjustment of age, gender, education, working status and alcohol consumption, the OR value ranged from 1.25 to 2.06. See Table 5.

**Table 5:** The proportion of people attempt to quit smoking among current smokers with and without TCD in a rural area of Shanghai, China

Tobacco related chronic diseases (TCD)		Attempt to quit		No intention to quit		OR	95% CI	OR^§^	95% CI^§^
		n	Proportion (%)	n	Proportion (%)				
Hypertension	Yes	371	14.83	2130	85.17	0.94	0.82–1.07	1.07	0.93–1.24
	No	746	15.68	4013	84.32	1.00		1.00	
Coronary diseases	Yes	41	18.81	177	81.19	1.28	0.91–1.82	1.46	1.02–2.07
	No	1076	15.28	5966	84.72	1.00		1.00	
Cerebral apoplexy	Yes	25	17.24	120	82.76	1.14	0.74–1.78	1.37	0.88–2.12
	No	1092	15.35	6023	84.65	1.00		1.00	
Diabetes	Yes	104	17.05	506	82.95	1.14	0.92–1.43	1.25	1.00–1.55
	No	1013	15.23	5637	84.77	1.00		1.00	
Chronic bronchitis	Yes	102	22.03	361	77.97	1.62	1.28–2.03	1.74	1.37–2.20
	No	1015	14.93	5782	85.07	1.00		1.00	
Asthma	Yes	27	22.50	93	77.50	1.61	1.04–2.49	1.67	1.08–2.58
	No	1090	15.27	6050	84.73	1.00		1.00	
COPD	Yes	9	25.71	26	74.29	1.91	0.89–4.09	2.06	1.01–4.47
	No	1108	15.34	6117	84.66	1.00		1.00	
Chronic gastritis	Yes	124	19.14	524	80.86	1.34	1.09–1.65	1.36	1.11–1.67
	No	993	15.02	5619	84.98	1.00		1.00	
Chronic hepatitis	Yes	49	19.07	208	80.93	1.31	0.95–1.79	1.32	0.96–1.82
	No	1068	15.25	5935	84.75	1.00		1.00	
Cancer	Yes	4	14.81	23	85.19	0.96	0.33–2.77	0.99	0.35–2.83
	No	1113	15.39	6120	84.61	1.00		1.00	

§: OR and 95% CI after the adjustment of age, education, gender, working status and alcohol consumption (covariates adjusted during the weighted logistic regression were selected by Directed Acyclic Graph (DAG) method)

## Discussion

Morbidity and mortality related to smoking is a major public health challenge worldwide [17]. In this study, the prevalence of smoking was 23.53%, with the prevalence of 57.48% for male and 0.30% for female. The prevalence of smoking in Songjiang district was lower than that in Jiangxi province (27.03% of adults were smokers) [9] and in China (66% of male and 3.1% of female were smokers in Chinese risk behavior surveillance) reported in 2012 [18]. For current smoking, in comparison with the migrant dynamics monitoring survey in China [10] in 2013 (54.40% of adult male and 3.70% of adult female were current smokers) and the GATS [19] in 2012 (the prevalence of current smoking was 52.90% for male and 2.35% for female), the prevalence was lower among Songjiang residents both for the male (48.36%) and the female (0.22%). The lower prevalence of smoking especially the current smoking might attribute to the implementation of new Shanghai Tobacco Control Regulation which was enacted in 2016 [20], and the public health education and intervention on smoking cessation in shanghai as well [11, 13]. According to the new Shanghai Tobacco Control Regulation, all public place in Shanghai is smoke free, and violators both of the smokers and the owner of public place will be punished by the government. Meanwhile, much more residents have noticed the physical harmfulness of tobacco use in recent years and stopped smoking successfully [21]. In this study, the prevalence of smoking cessation among Songjiang residents was 15.93%, which also explained the lower prevalence of current smoking. Whereas, we should also notice the high prevalence of current smoking in males, especially with an education of high school and aged 40–69 years old, we suggest tobacco control measures should be focused on this specific population in the future.

Hammond D, etc. [22] reported that tobacco use has became a serious public health problem globally and can increases the risk of many diseases. Tobacco smoking was a pathogenic causes for respiratory diseases, cardiovascular diseases, and many different form of cancers [8, 22]. Our study indicated that the prevalence of tobacco related chronic diseases among smokers ranged from 0.63 to 36.31%, and was obviously higher than non-smokers for hypertension, coronary diseases and chronic bronchitis, these findings were consistent with previous studies [23]. Tobacco contains carcinogenic substances which is harmful to human, tobacco consumption predisposes to many cancer, as well as hypertension, heart diseases, and other conditions [24]. In this study, we also noticed that the prevalence of all ten selected TCD among ex-smokers was higher than current smokers, that was because many smokers quit smoking after the diagnosis of the TCD, which lead to a high proportion of TCD patients among ex-smoker. And the elder age among ex-smokers could also be other explanation for the higher prevalence of TCD in this group.

Jha P [25] previously reported that smoking cessation had immediate and long term health benefits, it could decrease the occurrence of TCD and improve life quality of ex-smokers as well. Thus, many clinical guidelines have strongly recommended patients with TCD stop smoking to improve their health outcomes [26], but these recommendations have not effectively implemented by doctors to suggest smoking cessation among patients in China. In this study, we found that smokers with TCD had higher prevalence of quit smoking than smokers without TCD, over 50% of smokers quit smoking successfully after the diagnosis of TCD, and current smokers with TCD had higher smoking cessation attempt proportion than current smokers without TCD, this indicated that the occurrence of TCD even non-lethal one such as hypertension and diabetes could provide an opportunity for doctors to assist smokers to quit smoking. We suggest that all hospitals should implement clinical guidelines of smoking cessation among smokers when they visit a doctor, especially for TCD, which could increases the probability of success in smoking cessation. As Cho MH [27] reported that smokers might pursue a healthier lifestyle when diagnosed with chronic diseases, and if the doctors provided a smoking cessation suggestion, smokers were much more likely to attempt to quit smoking. But we should also notice that over 50% of ex-smokers quit smoking in 8 years after the diagnosis of cerebral apoplexy, diabetes, hypertension, cancer and coronary diseases, and took even longer time to quit smoking after the diagnosis of COPD, chronic gastritis, chronic bronchitis and asthma, these findings indicated that smoking cessation usually took a long time period and consume large resource and effort, so health related institutions should provide intense, repeated education about the adverse health effects and the benefits of quit smoking among smokers.

Previous studies identified age as one of the factors related to smoking cessation. In studies that conducted in Western countries, age was mostly reported as negatively related with smoking cessation [28]. Whereas, studies conducted in Asian counties usually found a positive association between age and smoking cessation [29, 30]. A study conducted in Korea reported that elderly smokers showed greater intention to quit smoking than younger smokers in the general population [27]. In this study, the prevalence of smoking cessation was higher among elder ex-smokers, and current smokers with older age had higher proportion of smoking cessation attempt, these findings were consistent with previous Asian reports [27]. But the discrepancy regarding age between Asian and Western countries might be induced by cultural and social differences; it is quite common in Asian including China for elders living with their younger generations, given the roots of Confucianism and its teachings of filial piety. In such living arrangements, it is likely that once diagnosed with a chronic illness, elderly patients would be strictly monitored and forced to lead a healthier lifestyle from their children, resulting in a positive relation between age and with smoking cessation.

A key strength in this study is the local HIS (Health Information System) verified TCD information. The HIS is a web-based system collecting disease information in real-time from hospitals of all levels in Shanghai. The HIS covers diseases information including but not limited to demographic information, onset of diseases, symptoms, diagnosis time, treatment, prescription information and cost, etc. In this study, we use the HIS to verify the self-report TCD information that collected by questionnaire for two reasons. First, the HIS verified TCD information is accurate without recall bias, it improves the study quality in the aspect of TCD prevalence calculation and the ascertainment of time interval between disease diagnosis and smoking cessation. Second, the consistency between HIS and the self-reported TCD information could reflect the data quality collected by the questionnaire, and the consistent rate in this study is finally proved as 95.88%. Meanwhile, we sampled 36, 698 residents that accounting for about 6% of total local population in Songjiang district, the large study population size is other strength of this study, study results could be generalized to total residents in Songjiang district, or even other rural areas of Shanghai.

There are some limitations in this study. First, the cross-sectional study design only allow the calculation of prevalence but not the incidence rate, which limited the estimation of causal relation between cigarette smoke and TCDs. Second, the time for TCD diagnosis was verified by the HIS, but the time for smoking cessation was self-reported by residents which could induce recall bias, leading to inaccuracy of time interval calculation between disease diagnosis and smoking cessation. Third, in this study, approximately 16% current smokers had smoking cessation attempt in a recent year but all relapsed, but reasons for smoking cessation relapse were not collected which impeded the establishment of targeted tobacco control measure in this group. Forth, this study was implemented in Songjiang district, a rural area of Shanghai, since smoking issue is not only a rural topic, we need select some urban residents in future studies. Fifth, for TCD diagnosis is correlated with age, it is better to describe the relationship between TCD diagnosis time and smoking cessation time with the adjustment of age, but due to the small number of ex-smokers with TCDs in this study, it is impossible to analyze the data by different age groups, which might induce some bias. Sixth, we only adjusted age, education, gender, working status and alcohol consumption to evaluate the role of TCD diagnosis in smoking cessation, the other features includes diseases severity, family relationships, marital status may also influence the study results as well, so incorporation of some improvements should be considered in further studies.

## Conclusions

The prevalence of current smoking was still very high among male residents in rural area of Shanghai, China, and the occurrence of TCDs even non-lethal one could provide an opportunity for doctors to assist smoking cessation among smokers.

## Data Availability

Data for this study can be made available upon request from the corresponding author. The request should state the title and aim of the research for which the data are being requested.

## Abbreviations

CD: Chronic Diseases
CI: Confidence Interval
COPD: Chronic Obstructive Pulmonary Disease
HIS: Health Information System
OR: Odds Ratio
SHS: Second Hand Smoke
TCD: Tobacco related Chronic Diseases
